# Primary Small Bowel Liposarcoma (Atypical Lipomatous Tumour) with Myogenic Differentiation

**DOI:** 10.1155/2010/807981

**Published:** 2010-07-26

**Authors:** J. Patel, R. Deb, W. Speake, T. A. MacCulloch

**Affiliations:** Department of Cellular Pathology, Nottingham University Hospitals, City Hospital Campus, NG5 1PB, and Royal Derby Hospital, Derby DE22 3NE, UK

## Abstract

Primary small intestinal liposarcomas originating in the small bowel are uncommon with a generally poor prognosis due to the advanced stage at the time of diagnosis. We describe a case of primary small bowel dedifferentiated liposarcoma presenting as a solid mass in the right iliac fossa. The current case is unusual as the tumour seemingly originated from the bowel and the well-differentiated component was seen extensively infiltrating the bowel wall including the small bowel submucosa.

## 1. Introduction

Primary small intestinal malignant mesenchymal tumours are uncommon, and liposarcomas originating in the small bowel are extraordinarily rare [1]. The early clinical symptoms of these malignancies are nonspecific and for this reason the disease is often diagnosed at an advanced stage. The prognosis of these lesions is generally poor owing to the diffusion of the disease at the time of diagnosis. Usually small bowel neoplasms are preoperatively identified only in 27–72% of cases and the percentage of surgical removal is from 65–80% in clinical literature [2]. We report the clinical, radiological, and pathologic findings of a primary small bowel dedifferentiated-type liposarcoma with divergent myogenic differentiation. 

## 2. Case Report

**Figure 1:**
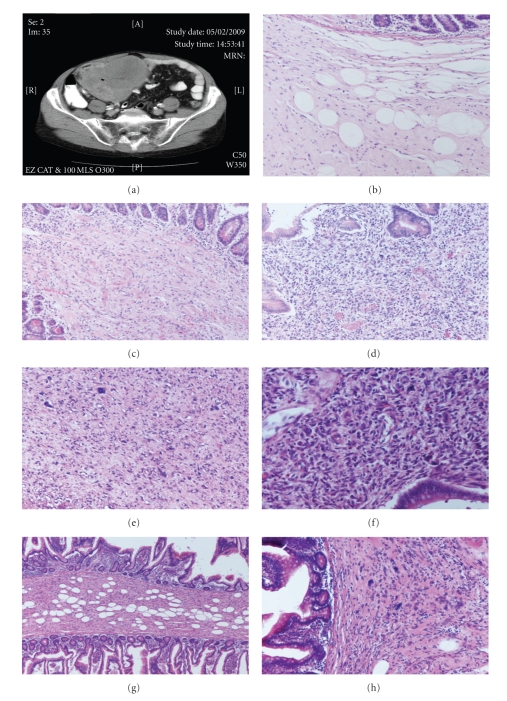
(a) A solid mass in the right iliac fossa not attached to the bowel wall, (b), (c) well-differentiated liposarcoma infiltrating the submucosa, (d), (e), and (f) dedifferentiated component infiltrating the small bowel, and (g), (h) well-differentiated liposarcoma at the small bowel resection margins.

A 59-year-old gentleman presented with a history of right iliac fossa mass. The CT scan showed a solid mass not obviously attached to the small or large bowel and had a possible fatty component at the edge (Figure 1(a)). The biopsy was reported as a malignant pleomorphic spindle cell lesion, which demonstrated strong positivity for SMA and desmin, whilst S100 and c-kit were negative. There was one mitosis per 10 hpf and no necrosis. Taking the radiological information into account, differential diagnosis of dedifferentiated liposarcoma with myogenic differentiation or a primary retroperitoneal leiomyosarcoma were suggested. Resection specimen received later comprised of a small bowel, the midpart of which was markedly distended by a pale mass measuring 14 × 11.5 cm. The nodular mass had a firm-cream yellow cut surface and approximately 60%–65% appeared to originate from the bowel wall. Several satellite lesions with similar macroscopic appearances were noted away from the main lesion. 

Histology showed the lesion to be composed of well-differentiated liposarcoma (atypical lipomatous tumour) with abrupt transition to areas with an extensive dedifferentiated component. The well-differentiated areas were represented by adipose tissue in which atypical adipocytes and lipoblasts were easily identifiable (Figures 1(b) and 1(c)). The dedifferentiated areas were represented by high-grade pleomorphic and spindle cell sarcoma that expressed diffuse strong SMA and desmin positivity indicating divergent myogenic differentiation (Figures 1(d), 1(e), and 1(f)). H-caldesmon was negative in the dedifferentiated areas. A diagnosis of dedifferentiated liposarcoma with myogenic divergent differentiation was therefore made. 

The tumour appeared to originate from the bowel and the well-differentiated component was seen extensively infiltrating the bowel wall including the small bowel submucosa. Critical examination of sections taken from the bowel wall well away from macroscopic tumour showed well-differentiated liposarcoma infiltrating along the submucosa. Furthermore well-differentiated liposarcoma was present at the resection margins (Figures 1(g) and 1(h)) despite these being at least 9 cms away from the macroscopic tumour.

## 3. Discussion

Dedifferentiated liposarcoma (DDLPS) is a term first introduced by Evans in 1979 [3] to describe liposarcomas containing a mixture of atypical lipoma/well-differentiated liposarcoma and a high-grade nonlipogenic sarcomatous component, the latter arising either within the same primary tumour (around 90% of cases) or in a recurrence (around 10% of cases). 

The nomenclature of the low-grade component of these tumours has been the subject of much confusion in the literature: the original terms “atypical lipoma” and “well differentiated liposarcoma” (WDLPS) pertain to homologous tumours arising in either the superficial or deep tissues, respectively. It was subsequently recognised that atypical lipomas and WDLPS have no metastatic potential without dedifferentiation and thus the nomenclature of these tumours was standardised in the WHO 2002 classification of soft tissue tumours. Currently all these lesions are termed atypical lipomatous tumours (ALTs) except when arising within the retroperitoneum or mediastinum. The term WDLPS was retained in these latter cases to emphasize that lesions at these particular sites are very difficult to eradicate, have a high rate of dedifferentiation, and often prove fatal. 

The risk of dedifferentiation occurring in ALT/WDLPS is higher in deep-seated tumours, particularly in the retroperitoneum (2/3 cases), and is probably a time-dependent phenomenon [4]. Dedifferentiation in ALT deep seated in the extremities is uncommon and in subcutaneous tissue is rare. 

Histologically, dedifferentiated liposarcoma is defined by the association of atypical lipomatous tumour/well-differentiated liposarcoma areas and a non-lipogenic high grade sarcoma, usually with an abrupt transition between the two components. Dedifferentiated areas exhibit a wide morphological spectrum. Most cases show areas of high-grade sarcoma resembling pleomorphic undifferentiated sarcoma or myxofibrosarcoma. In about five to 10% of cases, the dedifferentiated component shows divergent differentiation with a myogenic or osteochondrosarcomatous element. The genomic profile of DDLPS mirrors those of ALT and WDLPS, all of which show, on cytogenetic analysis, ring and giant chromosomes composed of amplified sequences of the 12q13-14 chromosome region. This amplicon is mainly composed of the MDM2 and CDK4 genes [5]. Amplification of MDM2 and CDK4 may therefore be responsible for the malignant tumour process. Dedifferentiated liposarcoma shows additional complex karyotypic abnormalities.

The most important prognostic factor in DDLPS is location with poor prognosis for retroperitoneal tumours, largely related to difficulty in achieving complete clearance of the tumour. Histological grade is not of prognostic value [6, 7]. The clinical course of dedifferentiated liposarcoma is mainly dominated by local recurrences (40%–60%). Metastatic potential is low (15%–20%) [3, 4] despite the tumour being a morphologically high-grade lesion. This is presumably a reflection of different molecular mechanisms of tumour development in these lesions as opposed to other high-grade sarcomas [5]. Indeed the rate of p53 mutation is lower than those of other high-grade sarcomas [8]. Interestingly, DDLPSs with myogenic divergent differentiation retain the low metastatic potential of conventional DDLPSs, significantly lower than that of comparable leiomyosarcomas [7]. Although WDLPSs/DDLPSs are the most common soft tissue sarcomas in the retroperitoneum [6], and while secondary involvement of the gastrointestinal tract can occur in cases of retroperitoneal sarcomas, liposarcomas primarily involving the gastrointestinal tract are extremely uncommon. Occurrence of these tumours in the small intestine [6] often presents a submucosal polyp or even intussusceptions. The current case is highly unusual in that it originated from, or extensively involved, the bowel wall including the submucosa, the latter primarily as the (presumably original) WDLPS component. Both ALT and WDLPS can recur at some distance from the original tumour (personal experience TM) and one of us (TM) has, on several occasions, seen two apparently unrelated fatty tumours prove to be a single ALT with synchronous presentation at some distance apart [6]. This phenomenon is good evidence that these tumours can be more extensive than clinically, radiologically, or even microscopically apparent because of the very close similarity of ALT and WDLPS, in areas, to normal adipose tissue. The current case demonstrates this phenomenon particularly well with the WDLPS component seen infiltrating extensively along the submucosa of the bowel wall. In the current case the risk of recurrence will not only be the around the surgical radial clearance margins but also at the bowel anastomosis site and further along the bowel itself.

## 4. Conclusion

In summary we have presented a very unusual case of dedifferentiated liposarcoma of the small bowel with myogenic divergent differentiation and extensive involvement of the small bowel submucosa. The case illustrates the particular point that this form of tumour may be much more highly infiltrative and extensive than apparent clinically and radiologically. This may go some way to explain the extremely high recurrence rates of these tumours, particularly in the abdominal cavity.
